# Exploring the Relationship between Innovative Work Behavior, Job Anxiety, Workplace Ostracism, and Workplace Incivility: Empirical Evidence from Small and Medium Sized Enterprises (SMEs)

**DOI:** 10.3390/healthcare8040508

**Published:** 2020-11-23

**Authors:** Madeeha Samma, Yan Zhao, Samma Faiz Rasool, Xiao Han, Shahzad Ali

**Affiliations:** 1 School of Management, Shanghai University, Shanghai 200444, China; madeeha@i.shu.edu.cn (M.S.); hanxiao2018@shu.edu.cn (X.H.); 2 Postdoctoral Station of Statistical, School of Innovation and Entrepreneurship, Entrepreneurship Institute, Guangzhou University, Guangzhou 510006, China; samma@i.shu.edu.cn; 3 School of Business and Economics, University of Management and Technology, Lahore 54000, Pakistan; 12004051007@umt.edu.pk

**Keywords:** innovative work behavior, workplace ostracism, workplace incivility, job anxiety

## Abstract

The aim of this study is to explore the relationship between workplace ostracism (WO), workplace incivility (WI), and innovative work behavior (IWB), using job anxiety as a mediating variable. Building on the conservation of resource (COR) theory, this study proposes a theoretical framework. In this framework, workplace ostracism and workplace incivility are theorized to strengthen innovative work behavior, directly and indirectly, through job anxiety. Data were collected from the workers of small and medium sized enterprise (SME) entrepreneurs located in Pakistan. To estimate the proposed relationships in the conceptual model, we used structural equation modeling (SEM) through AMOS-21. The outcomes of this study confirmed that workplace ostracism and workplace incivility had a negative impact on innovative work behavior. It was also confirmed that job anxiety mediates in the relationship between workplace ostracism, workplace incivility, job anxiety, and innovative work behavior. At the end of the study, we thoroughly discussed the conclusions, practical implications, limitations, and future research directions of the study.

## 1. Introduction

Workplace ostracism and workplace incivility influence innovative work behavior. Previous studies have discussed and debated it briefly [1,2]. However, the related job anxiety based on it still needs more discussion. These anxiety incidents occurring in employees’ lives affect their lives in different ways, such as their assigned work and family life. It also leads to a high psychological burden among employees. Some workers are mentally strong and dare to rise against these challenges, while others are mentally weaker, and they try to escape from these challenges and resign from their work to endorse workplace violence arising because of workplace ostracism (WO) and workplace incivility (WI). This paper is an effort to explain the intervening role of job anxiety between WO and WI and innovative work behavior to determine implications for practitioners and academicians.

People who interact with each other in the workplace may have different dimensions, which may be positive or negative. These different interactions lead to different outcomes. Positive interactions increase the productivity level in an innovative working environment, while negative interaction leads towards workplace violence resulting in decreasing innovative work behavior levels of workers and professionals [3]. Workplace ostracism and workplace incivility are not good for the workers, and it affects the performance, innovative work behavior, efficiency, and causes job anxiety among employees. It provokes a restless situation in organizational managers to measure their worker’s efficiency and organizational gain. Organizational managers put their efforts into examining human resource practices again after visualizing the factors creating WO and WI. A healthy working environment and innovative work behavior are achieved by reconsidering the human resource management practices by the organizational managers in the organization. In recent years, WO and WI have attracted the attention of practitioners because workers’ productivity levels are highly affected by WO and WI [1,4]. In terms of eliminating WO and WI and its effects on innovative work behavior, extant literature provides different thought-provoking insights, and it is very important for managers and researchers to find ways to solve this issue of a toxic workplace environment [5]. Workplace ostracism and workplace incivility affect innovative work behavior; measuring these behaviors requires more clarification by the heads of organizations because employees are considered the backbone of organizations. When there is a supportive workplace environment, it will increase the innovative work behavior of the employees; on the other hand, WO and WI decrease workers’ performance levels [3,5].

According to previous studies, workplace ostracism and workplace incivility decrease organizational performance and innovative work behavior [3,6,7]. This problem of performance affected by WO and WI got the attention of researchers to investigate and address this issue with higher consideration to identify the roots and causes of WO and WI for the organization, as well as for other stakeholders [8,9]. The performance of the individuals associated with entrepreneurs of SMEs located in Pakistan is affected by WO and WI. The direct relationship between WO and WI and job anxiety has been investigated in previous studies. But the relationship between WO and WI, job anxiety, and innovative work behavior has not yet been explored. Particularly job anxiety as an intervening role needs to be addressed and investigated by researchers. Many workers face ostracism and incivility at their workplace, but because of having a fear of being discriminated against, they do not disclose their worries. This affects their work efficiency, which also reduces their interest in their work and organization. Their work performance is also undermined because of the creation of a bad image that leads to a toxic workplace environment among peers and co-workers [10]. So, this research is an effort to be helpful for the entrepreneurs of SMEs of Pakistan to decrease WO and WI, and job anxiety to achieve innovative work behavior. This also will reveal how WO and WI affect employee’s work life, friends, and family life because WO and WI disturb the balanced work-life of workers resulting in a very disturbed, confused, and stressful life.

On the basis of the above-discussed literature, this study analyses the relationships between WO and WI, job anxiety, and innovative work behavior. Moreover, insights about WO and WI, job anxiety, and innovative work behavior are discussed. Thus, on the basis of the above-discussed literature, the research questions (RQ) addressed were as follows:

**RQ1:**  *How does workplace ostracism influence innovative work behavior*?

**RQ2:**  *How does workplace incivility influence innovative work behavior*?

**RQ3:**  *How does job anxiety intervene between workplace ostracism, workplace incivility, and innovative work behavior*?

## 2. Literature Review

### 2.1. Workplace Ostracism

Experiencing isolation in the workplace and having feelings of not being part of the organization because of workplace ostracism [11,12,13] leads to workers showing less involvement and interest in their work and also to show dissatisfaction with their work [10]. Workplace-ostracism produces counter-productive work behavior [14]. Workplace ostracism is a factor that brings with it stressful behavior, emotional, physical, and mental exhaustion, and less productive behavior [15]. Workplace ostracism affects employees psychologically and physically, which results in job anxiety and disrupts innovative work behavior [16]. Demotivation arising because of workplace ostracism among employees affects the efficiency and innovative work behavior of the workers as well as making an organization or firm less efficient.

### 2.2. Workplace Incivility

Workplace incivility is different from other negative behaviors experienced by the employees from their co-workers. These negative behaviors are called deviations from positive natural behaviors [17]. Intentionally or unintentionally, incivility instigators may be harmful to the workers of an organization in order to benefit from it. Cracking a rude joke to co-workers may result in a form of humiliation for another worker [18]. The intention in this situation is ambiguous. The person cracking the joke may have a bad sense of humor, or maybe the intention behind the joke was to humiliate the co-worker [19,20,21]. Abusing non-verbally or verbal actions and attitudes towards co-workers are counted as incivility in the workplace [22]. The focus of entrepreneurs in SMEs, management scientists, and academicians is to eradicate and reduce the basics roots causing workplace incivility in which workers tend towards sacrificing their self-respect, job satisfaction level, and their work productivity and efficiency. It is also socially harmful and stressful for the business entity, as it results in a less progressive business [23]. These deviations result in a bad image for the co-workers at the workplace.

### 2.3. Job Anxiety 

Job anxiety is defined as “an unpleasant emotional state characterized by concerns, fright, distress, and restlessness that is a response to perceived physical and/or psychological danger” [24] and is experienced in a state of threat to valued resources [25]. Furthermore, job anxiety can also be a result of exceeding demand for the job from an employee [26]. 

### 2.4. Innovative Work Behavior

A behavioral series that allows employees to think in a creative way for optimization of work performance and its procedure and routines is known as innovative work behavior. These behavioral manifestations usually involve the identification of work-related problems, the introduction of innovative and better ideas, and the implementation of those ideas, etc. However, innovative work behaviors are somewhat different from employee’s creativity focused on discovering and generating ideas [27], as creativity focuses on processes to initiate the relatively latest and better ideas [28]. When compared to creativity, innovative work behavior (IWB) has more focus and purpose as it includes the identification, analysis, design, implementation, and evaluation of new ideas and links it with improvement in the work process and resulting performance. 

Hence, it can be said that creativity is a sub-dimension of IWB based on its role in the initial phase of gap identification to improve performance and the role in proposing creative new ideas [29]. It has been found that the scope of IWB is even bigger than the constructs of productivity, i.e., productive working behavior [30] and personal initiatives [31] that are focused on the identification and implementation of new ideas by an individual in a productive way.

The constructs of pro-activeness highlight the tendency of an individual to implement the ideas actively; however, it does not involve innovative idea generation [32]. Therefore, the concept of IWB is aimed at generating and implementing innovative new ideas purposefully, which have significant importance for an organization, especially to improvise the user experience, the development of product design, and procedural optimization.

Ongoing research work uses expectancy theory for guidance while discussing expected IWB based on the mechanisms that drive innovation [33]. Research work has been conducted on highlighting the internal factors which drive IWB, and less focus has been placed on discussing the negative external factors influencing IWB [34]. Hence, this study investigates the relatively unexplored area of external workplace factors, such as workplace incivility and workplace ostracism, its impact on IWB, and also attempts to investigate the indirect effects of job anxiety [35,36,37].

### 2.5. Conservation of Resource Theory (COR) Theory

Conservation of resource (COR) theory was used in the study to support and substantiate our theoretical predictions. Employees exposed to a destructive workplace environment tend to have a more negative work attitude by showing less interest in the assigned work as compared to a cooperative workplace environment. On the basis of these negative behaviors, workers also experience a depletion of resources. It indicates an afterward motivation in the conservation of resources in work-related efforts [38]. Conservation of resource theory explains that employees do not show interest in a positive work attitude when experiencing work-family conflict [39] or dysfunctional politics within the organization [16]. Similarly, we argue that resource loss can occur because of employee WO and WI exposure. These losses may be in the form of hurting their self-respect and self-absorption around the functioning of their organization in which they work [40], such that they stop caring about the wellbeing of their co-workers and try to conserve energy to recover those losses [41]. Employees having the ability to regain the lost resources and play a role in protecting the remaining resources are very suitable in resisting the negative consequences arising because of toxicity in the workplace environment [42].

Moreover, conservation of resource theory and its basic idea revolving around resource loss [43] suggest that employee’s perceptions of workplace difficulties and hurdles can create bad and dangerous effects. These harmful perceptions of employees who are exposed to these conditions are of such a level that they play a role in altering personal characteristics, or operating in such an environment can aggravate their experience of losing resources. For instance, in the presence of a political, organizational climate, employee job performance is reduced and decreased because of experiencing an unfair provision of the information [44]. Similarly, we propose that anxiety or depression related to work is more strong in male employees as compared to their female co-workers. This anxiety is because of the indirect effect of the WI depersonalization among the employees. Male employees with higher education are exposed to more of this anxiety as compared to those employees who do not have a high education level [45,46]. According to our predictions, especially in the empirical context of this study in Pakistan, when male employees with high education levels are treated with disrespect, they face more losses of their personal dignity. Because in the Pakistani strict education culture, males are more dominant as compared to females [47]. Thus, workplace incivility increases job anxiety and depersonalization among employees.

Formally, we propose that anxiety in jobs and increases in the perception of losing resources occurs because of workplace incivility, causing co-worker depersonalization [41]. These feelings of anxiety, which employees experience during their work, create worries about their organizational functioning and completion of tasks that are assigned to them [48]. Employees that believe that their co-workers hurt their self-respect and feelings result in having concerns about their job situation; this situation leads employees not to care about their work and the wellbeing of their workers because of this dehumanizing behavior [49,50]. Previous research has proved that workplace incivility decreases the reserving of positive energy, but it has not been examined by researchers how much this affects the energy in the form of job-related anxiety and depersonalization among employees [51,52,53,54]. Thus, the role of job-related anxiety, which is a very important research problem because of the resource-draining, WI, and depersonalization towards co-workers among male and highly educated workers, is the main importance of this study. Male and highly educated workers should also have relevance to other countries that have a similar culture to Pakistan. 

## 3. Hypotheses Development

### 3.1. Workplace Ostracism and Innovative Work Behavior

Workplace ostracism shows a negative direct relationship with IWB. Previous studies indicate that when employees feel they are ignored and are not part of the conversation, the group becomes demoralized and feels uncounted in the organization. This affects the ostracised employee’s ego, confidence, and productivity, which leads to inefficient and less innovative work behavior. Continuously being ostracized make an employee feel like a low valued employee [55]. Employee’s self-esteem is affected because of ostracism. When employees feel being ignored and hurt, they start losing interest in their assigned work, which ultimately leads them to less IWB [56]. There is a negative relationship between ostracism and innovative work behavior. If workplace ostracism is high, then worker innovative work behavior is reduced; when workplace ostracism is low, then worker innovative work behavior levels are higher. Thus, according to the above-discussed literature, this negative relationship between ostracism and innovative work behavior is shown as the following hypotheses.

**Hypothesis** **1.** *Workplace ostracism negatively influences innovative work behavior.*

### 3.2. Workplace Incivility and Innovative Work Behavior

Workplace incivility has a direct relationship with innovative work behavior. This is a direct negative relationship between workplace incivility and innovative work behavior. Studies previously done show the relationship between workplace incivility and innovative work behavior and indicate that employees who are exposed to incivility in the workplace feel disrespect, lose their dignity and self-respect; this kind of behavior leads to them being less efficient and less productive, which ultimately results in low productivity levels, which is not good for the employees as well as for the organization in which those employees are working [57]. Impoliteness being faced by the employees make them feel aggression, which affects their innovative work behavior level, resulting in low worker productivity [58]. There is a negative relationship between workplace incivility and innovative work behavior. Thus, according to the above-discussed literature, if the workplace incivility is higher, the innovative work behavior level will decrease; if the workplace incivility is low, the productivity level of the workers increases. This relationship among workplace incivility and innovative work behavior resulted in the following hypothesis:

**Hypothesis** **2.** *Workplace incivility negatively influences innovative work behavior.*

### 3.3. Mediating the Effect of Job Anxiety 

**Figure 1:**
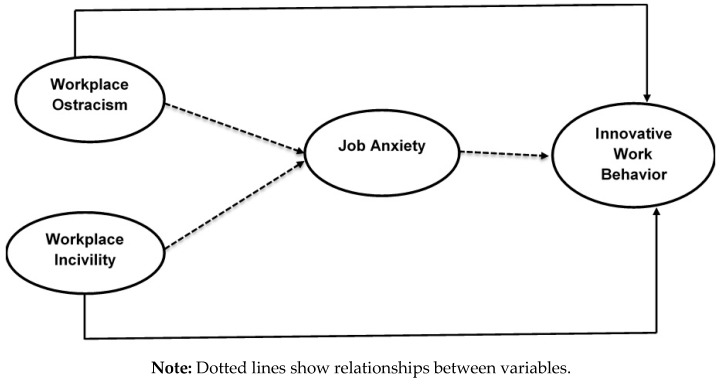
Proposed research model.

There has been a lot of literature on job anxiety, indicating its relation with workplace ostracism along with workplace incivility [59]. The COR theory postulates that anxiety is caused by factors that may lead to an actual or potential threat to the organization’s valuable resources [60]. It has been found that it negatively affects intangible and intangible resources such as self-esteem, confidence, and mastery, etc. Prior studies indicate that job anxiety mediates between workplace ostracism, workplace incivility, and innovative work behavior [61,62]. These relationships demonstrate that if employees in entrepreneurial SMEs are treated with disrespect, then they will lose innovative behavior [63]. COR theory further adds that rude behavior between employees can make them angry, stressed, anxious, and may further lead to reduced job performance [64,65]. Disrespectful behavior toward employees from colleagues and organizations can further lead to depleted energy on the job [66] and results in the inability to meet their job-related performance expectations [53,67]. Hence, if the job anxiety reaches a higher level, it may well be because their colleague failed to show respectful behavior towards their feelings and dignity. Taken together, these arguments suggest that employees’ job-related anxiety mediates workplace ostracism, workplace incivility, and innovative work behavior. Figure 1 summarizes the proposed research model of this study.

**Hypothesis** **3a.** *Job Anxiety mediates between Workplace ostracism and innovative work behavior.*

**Hypothesis** **3b.** *Job Anxiety mediates between Workplace incivility and innovative work behavior.*

## 4. Research Methodology 

### 4.1. Research Approach

The survey approach, based on an empirical questionnaire, was adopted in this research. The questionnaire design and data collection was based on the hypotheses above and started with the help of a quantitative method that was followed by a descriptive or inferential application. Questionnaire surveys are a popular and extensively used research technique for quick collection and analysis of data from a target population [68,69]. 

### 4.2. Questionnaire Designing 

The purpose of this study was to determine how WO and WI, directly and indirectly, influence innovative work behavior, using job anxiety as a mediating variable. All of the items in the latent variables were measured using a five-point Likert scale (see Appendix A). A pilot study was conducted to check the reliability and validity of the questionnaire. For the pilot study, we selected ten academic professors and ten SME entrepreneurs (who were aware of the topic of this study) to review the questionnaire. Their feedback led to several changes in item wording and the final version of the survey. To check the face validity of respondents, the study refined the questionnaire wording, assessed logical consistencies, judged the ease of understanding, and identified areas for improvement. Overall, the questionnaire was regarded as concise and easy to complete. The revised questionnaire was distributed among the selected population. All items that we used in the questionnaire are given in Appendix A.

### 4.3. Variables Measurements 

Two independent variables (WO and WI), one mediating variable (job anxiety), and one dependent variable (innovative work behavior) were used in this study. The items of workplace ostracism were adopted from [3,6]. All items of workplace ostracism were measured with the five-point Likert-Scale (1: “strongly disagree”; 5: “strongly agree”). Sample items included: “My supervisor/co-worker/subordinate always ignored me at work” and “My supervisor/co-worker/subordinate during the conversation shut at me”. The alpha of WO was 0.812. The items used in the study were considered valid because of their alpha value above the standard 0.70.

The items of workplace incivility were adopted from [3,6]. All items were measured with the five-point Likert-Scale (1: “strongly disagree”; 5: “strongly agree”). Sample items included “I knew what has to be done, so I doubled your efforts to make things work” and “I often talk to someone who could help me with the situation”. The alpha of WI was 0.821. The standard value of alpha is 0.70 and higher. So, the items we used in this research instrument were valid.

The items of mediating variable job anxiety were adopted from [39]. For the measurement of job anxiety, we applied the five-point Likert-Scale (1: “strongly disagree”; 5: “strongly agree”). Sample items included: “At work, my feeling are down, anxiety, and hopeless” and “I have a bad feeling about myself—e.g., I am a failure or have let myself or my family down”. The results are indicating the 0.859 alpha value of job anxiety. The standard value of alpha is 0.70 and higher. So, the items we used in this research instrument were valid.

We used “employees innovative work behavior” items developed by Sethibe and Steyn [70]. All items were measured on the five-point Likert Scale (1: “strongly disagree”; 5: “strongly agree”). Sample items included: “I always search out new technologies, processes, techniques, and/or product ideas” and “I feel that my supervisor/co-worker/subordinate are more efficient to me”. Results are indicating the 0.767 alpha value of IWB. The standard value of alpha is 0.70 and higher. So, the items we used in this research instrument were valid.

### 4.4. Sampling and Data Collection

The data were collected from the workers of entrepreneurial SMEs located in Pakistan. This research was conducted in 2020, and the aims of the study were introduced to all respondents at the start of the questionnaire in the guidelines drafted; moreover, according to the ethical rules of research; respondents had been told that their provided information will not be revealed to anyone and will solely be used for research purposes. The respondents were chosen using a convenience sampling method. There were two main reasons to select convenience sampling. First, it was easy to use. Second, in a pilot study, convenience sampling is usually used because it allows the researcher to obtain necessary data and trends regarding their study without the complications of using a randomized sample. The survey was conducted in four cities in Pakistan: Islamabad, Karachi, Lahore, and Sialkot. The authors selected these cites because most SME entrepreneurs from these cities exercise modern innovation practices and have incorporated modern innovation mechanisms in their business cycles. The authors collected online data from the target respondents. Anticipating a relatively lower response rate a total of 360 questionnaires were distributed, and 260 questionnaires were received. About six questionnaires were eliminated because of incompletion. A total of 254 respondents were considered from further analysis, and the overall response rate was 70%. The detail of the demographics in this study is presented in Table 1.

## 5. Analysis and Results

### 5.1. Reliability Analysis

As mentioned in the sampling section, the scales were tested for their reliability, and the analysis for reliability was conducted using SPSS-21. The value of alpha was taken as the indicator of scales reliability. The standard value of alpha is 0.70 and higher. Table 2 of this study shows that the scales used were highly reliable as their Cronbach’s alpha values were above the standard 0.70. Another indicator used to assess the reliability of each item was the corrected-item correlation value. 

### 5.2. Descriptive Statistics

The descriptive analysis was conducted to find out the descriptive characteristics of the data. Values in Table 3 revealed that the mean values ranger from 2.0 to 3.9, while the Standard Deviation ranged from 0.59 to 0.68.

### 5.3. Regression Analysis

Regression analysis of this study was performed using the AMOS 21 to examine the directional dependence of the variables. AMOS uses variance-based structural equational modeling, which is not only used to check the conceptual model fitness but is also used to validate the structural model for regression analysis [71,72]. Table 4 shows the values of direct and indirect effects of two constructs of the study (workplace incivility and workplace ostracism) on innovative work behavior and the indirect effect of job anxiety on the above-mentioned relationships. The results of the analysis showed a significant negative effect on workplace incivility of innovative work behavior with IWB (β = −0.598, *p* < 0.05), which supports Hypothesis 1 of the study. The results also revealed that there was a significant negative effect of workplace ostracism on innovative work behavior with IWB (β = −0.773, *p* < 0.05), which supports Hypothesis 2 of the study. Furthermore, the results of indirect effect of job anxiety mediated between workplace incivility (β = 0.139, *p* < 0.05) and workplace ostracism (β = −0.077, *p* < 0.05). this supports Hypotheses 3a and 3b.

## 6. Discussion

Workplace ostracism and workplace incivility have attracted the attention of many researchers. A co-worker environment keeps employees in a confident and relaxed state from which they can achieve their maximum output, whereas a WO and WI create job anxiety. An organization suffering from WO and WI is the main source of job anxiety for employees.

First, the results of this study show that WO is directly negatively linked with innovative work behavior. This supports Hypothesis 1, in which higher levels of workplace ostracism lead to a low level of innovative work behavior. This result is also in line with the COR theory [73]. Based on trust and honesty, organizations, employees, and stakeholders can be considered as cooperating, but sometimes the relationships become unfit because of workplace ostracism. So, findings suggest that WO brings job anxiety, depression, and insomnia among employees and affects their innovative work behavior [74,75,76].

Secondly, the outcomes of this research show that there is a negative relationship between WI and IWB. A high level of WI among employees in the workplace is likely to produce less innovative work behavior. This supports Hypothesis 2, in which higher levels of WI lead to lower levels of IWB [67]. Similarly, Rasool et al. [3] examined 180 workers employed at Chinese banks, and the findings of their research showed that workplace incivility is directly negatively linked with innovative work behavior. These findings are also in line with the COR theory and RBV (resource-based-view) theory [6,67,73]. This is consistent with prior studies that have shown a negative relationship between WI and IWB [7,77].

Thirdly, the findings of this study also indicated that job anxiety is mediated by the relationship between workplace ostracism, workplace incivility, and innovative work behavior, which supports the proposed Hypotheses 3a and 3b. Moreover, previous studies also support the outcomes of this study [78,79]. COR theory also supports the results of this study [39,67]. Prem et al. [80] argued that due to job anxiety, employees could not perform well at the workplace, which also affects their personal life. So, the employees who are facing job anxiety have headaches, and they cannot sleep well. De Clercq et al. [39] conducted a study in Pakistani public and private sector telecom organizations. The results also support our study and suggest that job anxiety is a critical mechanism by which workplace incivility and workplace ostracism causes employees to withdraw from their immediate work environment and results in the dehumanization of co-workers.

## 7. Limitations and Future Research

This research was conducted to fill the literature gap in the related area. The practical contribution of this research is to help organizations, especially entrepreneurial SMEs, to critically understand the factors of success that have been relatively unexplored. Despite the results, there are a few limitations associated with this research that might affect the interpretation of results. The first limitation was that the respondents were selected only from one country (Pakistan). This is a limitation in terms of generalizability under the influence of cultural and contextual biases. The second limitation was the sample size of the study, which may influence the generalizability of the results. However, to overcome these limitations, the research has undertaken used certain precautions. To eliminate the cultural and contextual biases, the results of the research have been interpreted in line with the relevant studies, and to further improve the study, a pilot study was conducted beforehand so that the questions were clear to the respondents. 

## 8. Conclusions 

This study offers some major contributions to the existing literature by testing the concepts developed in a western set in a non-western culture. This is the first time that research has demonstrated, against most theoretical expectations, a potentially negative influence on innovative work behavior from the presence of workplace violence. The findings of this study summarized as follows: The results for entrepreneurial SMEs, especially in the context of Pakistan, indicate that when the employees feel to be ignored and are not being a part of the conversation, and the group become demoralized and feel they are not counted in the organization. This affects the ostracised employee’s ego, confidence, and productivity, which leads to inefficient and less innovative work behavior. Thus, workplace incivility increases job anxiety and depersonalization among the employees. Moreover, COR theory has proven through this study that anxiety is caused by factors that may lead to an actual or potential threat to the employee’s valuable resources. It has been found that it negatively affects intangible and intangible resources such as self-esteem, confidence, mastery, etc. This research was based on the COR theory, and the results of the current study prove that the COR theory supports the above-mentioned relationship. Prior studies on workplace violence also indicate that workplace ostracism and workplace incivility are the critical factors that affect the short-term performance of SMEs. Similarly, innovative work behavior is a critical factor that enhances the short-term and long-term performance of SMEs, which brings sustainability to organizations. By discussing the impact of workplace ostracism and workplace incivility on innovative work behavior, this study reveals the potential long-term negative effects of workplace ostracism and workplace incivility on enterprises.

## Figures and Tables

**Table 1:** Sample characteristics.

Measure	Items	Frequency (*n*)	Percentage (%)
Gender	Male	212	83.5
	Female	42	16.5
Experience	<5 years	93	36.6
	6–10 years	115	45.3
	>10 years	46	18.1
Position	Senior manager	73	28.7
	Middle manager	138	54.3
	Administrative staff	43	16.9
Education	Graduate	162	63.8
	Undergraduate	92	36.2

**Table 2:** Construct Reliability and Correlation.

Construct	Reliability	Correlations			
	Alpha	WO	WI	JA	IWB
Workplace Ostracism	0.812				
Workplace Incivility	0.821	0.385 **			
Job Anxiety	0.859	0.246 **	0.646 **		
Innovative Work Behavior	0.767	−0.823 **	−0.572 **	−0.282 **	

Note: ** Significant at the 0.05 level. WO: Workplace Ostracism; WI: Workplace Incivility; JA: Job Anxiety IWB: Innovative Work Behavior.

**Table 3:** Descriptive characteristics.

Construct	Minimum	Maximum	Mean	SD
Workplace Ostracism	1.00	4.07	2.33	0.66
Workplace Incivility	1.11	4.22	2.18	0.59
Job Anxiety	1.00	5.00	2.09	0.68
Innovative Work Behavior	1.83	5.00	3.89	0.62

Note: SD: Standard deviation.

**Table 4:** Regression weights.

Direct Effect						
Hypotheses 1 and 2			Estimate	S.E.	C.R.	*p*-Values
IWB	←	WI	−0.598	0.054	−11.097	0.000
IWB	←	WO	−0.773	0.034	−23.064	0.000
**Indirect Effect**						
**Hypothesis 3a**						
JA	←	WI	0.735	0.055	13.464	0.000
IWB	←	WI	−0.699	0.07	−10.013	0.000
IWB	←	JA	0.139	0.061	2.257	0.024
**Hypothesis 3b**						
JA	←	WO	0.252	0.062	4.037	0.000
IWB	←	WO	−0.754	0.034	−22.021	0.000
IWB	←	JA	−0.077	0.033	−2.314	0.021

Note: JA: job anxiety; WI: workplace incivility; IWB: innovative work behavior, WO: workplace ostracism; S.E.: standard error; C.R.: composite reliability.

## Appendix A. Research Instrument

	**Workplace Ostracism**
1.	My supervisor/co-worker/subordinate always ignored me at work.
2.	My supervisor/co-worker/subordinate is not answer my greeting.
3.	My involuntary sat alone in a crowded lunchroom at work.
4.	My supervisor/co-worker/subordinate avoided me at work.
5.	My supervisor/co-worker/subordinate during the conversation shut at me.
6.	My supervisor/co-worker/subordinate refused to talk to me at work.
7.	My supervisor/co-worker/subordinate at work treated me as if I am not a part of the organization.
	**Workplace Incivility**
8.	I just concentrated on what I will to do the next step.
9.	I knew what has to be done, so I doubled your efforts to make things work.
10.	I drew on your past experiences, and I have similar encounters before.
11.	I came up with a couple of ways of handling the situation.
12.	I practiced confronting the person with family, friends, others.
13.	I often asked my relative or friend for advice.
14.	I often talk to someone who could help me with the situation.
	**Job Anxiety**
15.	I have little interest or pleasure in doing things.
16.	At work my feeling are down, anxiety, and hopeless.
17.	I feel trouble falling or staying asleep, or sleeping too much.
18.	I feel tired or having little energy.
19.	Most of the time I feel poor appetite or overeating.
20.	I have a bad feeling about myself—e.g., I am a failure or have let myself or my family down.
	**Innovative Work Behaviour**
21.	During the past six months, my actual innovative work performance is decreasing day by day.
22.	I always search out new technologies, processes, techniques, and/or product ideas.
23.	During the past six months, most of the time I think about the lousy productivity of my job.
24.	I feel that my supervisor/co-worker/subordinate are more efficient to me.
25.	I feel that my tasks are more challenging than my co-workers.
26.	I don’t sleep well, which effect my work productivity.
27.	During the last six months, I am feeling that I am getting old.
